# Validation of the Social support and Pain Questionnaire (SPQ) in patients with painful temporomandibular disorders

**DOI:** 10.1186/s10194-017-0766-6

**Published:** 2017-05-22

**Authors:** Songlin He, Jinhua Wang

**Affiliations:** 1 College of Stomatology, Chongqing Medical University, No. 426 Songshibei Road, Chongqing, 401147 China; 2 Chongqing Key Laboratory of Oral Diseases and Biomedical Sciences, Chongqing, China; 3 Chongqing Municipal Key Laboratory of Oral Biomedical Engineering of Higher Education, Chongqing, China

**Keywords:** Validation, SPQ, Temporomandibular disorders

## Abstract

**Background:**

The present study aimed to validate of Social support and Pain Questionnaire (SPQ) for use in Chinese patients with painful temporomandibular disorders (TMD).

**Methods:**

The Chinese version of SPQ was produced by translation and cross-culturally adaptation of the original English version according to international guidelines. The Chinese version of SPQ was then distributed to a total of 118 patients with painful TMD. Reliability of the SPQ was evaluating using internal consistency and test-retest methods and validity of the SPQ was determined by construct validity and convergent validity. The exploratory factor analysis (EFA) was used to assess the construct validity of SPQ. And convergent validity was assessed by correlating the SPQ scores with the score of a global oral health question.

**Results:**

The Chinese version of SPQ has a high internal consistency (Cronbach’s alpha value, 0.926) and good test-retest reliability ((intraclass correlation coefficient (ICC), 0.784). Construct validity was evaluated by EFA, extracting one factor, accounting for 74.8% of the variance. All factor loadings of the six items had exceeded 0.80. As regards convergent validity, the SPQ showed good correlation with the global oral health question.

**Conclusion:**

These findings support that the Chinese version of SPQ can be used as a reliable and valid tool for Chinese patients with painful TMD.

## Background

Temporomandibular disorders (TMD) are “a group of biopsychosocial illnesses characterized by chronic painful conditions and dysfunction in the muscles of mastication and the temporomandibular joint” [1]. It affects 14.9 to 17.9% of Chinese population [2, 3]. Pain is one of the most common clinical symptoms of TMD [4]. TMD pain involves not only the masticatory muscles and TMD, but also affects the adjacent structures such as ears, teeth, head and neck muscles [5]. Studies have shown that oral health-related quality of life (OHRQoL) is negatively affected by TMD pain [6–8].

In recent years, there is a growing interest in exploring the relationship between social support and pain behavior [9–11]. Several studies reported that social support plays an important role in overcoming pain-related disability [9, 12]. Other studies, however, have shown that social support has negative effects on mobility and physical function [13, 14]. To further explore the impact of social support on chronic pain may give us new insights into the treatment of pain-related diseases [15]. Most measures on social support are mainly concerned with social support in daily-life situations, rather than its relationship to pain [16, 17]. The West Haven-Yale Multidimensional Pain Inventory (MPI) is the widely used instrument to evaluate the chronic pain experience from the cognitive-behavioral perspective [18, 19]. It does concern support related to pain by one person. However, it fails to capture the social support from others, like friends and family [20].

Recently, the Social support and Pain Questionnaire (SPQ) was developed by the Academic Centre for Dentistry Amsterdam (ACTA) to measure satisfaction with social support related to pain [15]. It is a 6-item, one-factor structure, self-administered English instrument [15]. The SPQ is a reliable and valid tool to evaluate the patient’s satisfaction with pain-related social support [15].

To make this instrument suitable for other languages and cultures, an internationally standardized evaluation procedure must be implemented. Therefore, the aim of our study is to validate a Chinese translation of the SPQ for patients with painful TMD.

## Methods

### Patients

A total of 118 consecutive patients were recruited from our university clinic between January 2015 and October 2015. The inclusion criteria were: at least 18 years of age, a diagnosis of painful TMD according to the Research Diagnostic Criteria for Temporomandibular Disorders [21], complaint of pain for at least 3 months, and sufficient ability to fill in the questionnaire. The exclusion criteria included: had a history of psychiatric illness, had acute pain for less than 3 months, a systemic disease, dental pain, and were reluctant to sign informed consents. For the sample size, it was determined according to the quality criteria for health status questionnaires [22]. The criteria suggested that a study should recruit at least 100 patients for reliability and validity analysis [22]. Before completing the SPQ, patients were instructed how to fill in the questionnaire. When necessary, they can consult interviewer at any time.

All the patients signed the written informed consent, and the present study was approved by the Ethics Committee of Chongqing Medical University.

### The SPQ

The original SPQ is composed of 6 items: *the support that I get from the people around me, the advice that I get from the people around me, how much opportunity I have to discuss the pain with the people around me, how much care I receive, how much understanding the people around me show and the practical help people around me give.* The response is scored using a Likert scale from 0 to 4 (0 = very dissatisfied, 1 = dissatisfied, 2 = neutral, 3 = satisfied, 4 = very satisfied). In general, higher scores indicate more satisfaction with social support related to pain. Additionally, to assess the convergent validity, an extra global question ("*In general, how would you rate your social support related to pain* ") was added at the end of instrument. The question is scored with the following options: (1 = very good, 2 = good, 3 = fair, 4 = poor, 5 = very poor).

### Translation and cross-cultural adaptation

The process of translation and cross-cultural adaptation of the original SPQ was carried out according to Guillemin’s guidelines [23].

1. Translation into Chinese: the original SPQ was firstly translated into Chinese by two bilingual translators independently. One translator was a linguist and the other one was a clinical psychology expert.
2. Back translation into English: the two other bilingual translators who had no knowledge of the original SPQ translated the Chinese version into English. They were native English speakers and fluent in Chinese.
3. Expert committee compared the two versions: the two versions were compared and assessed by an expert committee (two public health experts and two dental experts) to assure that there were no differences in the meaning of the items. The initial Chinese version of SPQ was then obtained.
4. Pilot test: a pilot test of the initial Chinese version of SPQ with 20 painful TMD patients was carried out to ensure that the formulation of items was clear. The expert committee reevaluated all of findings and then approved the final Chinese version of SPQ.

### Statistical analysis

#### Reliability

A Cronbach’s alpha between 0.70 and 0.95 and an Intraclass Correlation Coefficients (ICC) value over 0.70 were used to determine the internal consistency and test-retest reliability, respectively [22]. The ICC vales were analyzed by asking 30 patients to complete the Chinese SPQ again after a period of 2-week.

#### Validity

Validation of the Chinese SPQ included the assessment of the construct validity and convergent validity.

Construct validity was examined using exploratory factor analysis (EFA). The Kaiser–Meyer–Olkin test and Bartlett’s sphericity test were firstly carried out to determine if the data was suitable for performing the EFA [24]. The EFA followed by varimax rotation method was carried out to evaluate the dimensionality of the Chinese SPQ. Significance was defined as a loading higher than 0.40.

Convergent validity was assessed by correlating the SPQ scores with the score of the global oral health question. The correlation values were categorized as follows: poor correlation (0–0.20), fair correlation (0.21–0.40), moderate correlation (0.41–0.60), good correlation (0.61–0.80), and excellent correlation (0.81–1.0) [25].

All the statistical analysis were performed using version 20.0 of the SPSS software (IBM Corp. 2011; NY; USA). In the present study, the *p* value of <0.05 was considered statistically significant.

## Results

### Patient characteristics

A total of 118 patients with painful TMD were recruited from a university-affiliated dental clinic. All the patients declared that the questions were easy to understand and completed the questionnaire fully. Patient characteristics are shown in Table 1. Of the 118 patients selected, 56.8% were female, with the mean age of 46.4 ± 14.7 years old. The majority of patients were employed (72.9%), and half of them had been educated in middle school (54.2%). The pain were classified into joint pain (32.2%), muscle pain (36.4%) and mixed pain (31.4%). The mean scores, corrected item-total correlations and Cronbach’s alpha if item deleted results for the SPQ are presented in Table 3.

**Table 1:** Characteristics of patients (*n* = 118)

		Value
Age (years)	Mean (SD)	46.4 ± 14.7
Gender (n)	Male	51 (43.2%)
	Female	67 (56.8%)
Employment (n)	Employed	86 (72.9%)
	Unemployed	32 (27.1%)
Education history (n)	Primary School	25 (21.2%)
	Middle school	64 (54.2%)
	Bachelor degree or above	29 (24.6%)
Classification of pain (n)	joint pain	38 (32.2%)
	muscle pain	43 (36.4%)
	mixed pain	37 (31.4%)

### Reliability

As displayed in Table 2, Cronbach’s alpha for the SPQ was 0.926. The test-retest reliability of the instrument was also acceptable, with the ICC value of 0.87 (Table 2).

**Table 2:** Internal consistency, test–retest reliability and convergent validity of the SPQ

Scale	Internal consistency (*n* = 118)	Test–retest (ICC, 95% CI) (*n* = 30)	*r*_s_* (95% CI) (*n* = 118)
Total score	0.926	0.784 (0.648–0.893)	0.624 (0.554–0.719)**

*ICC* Intraclass correlation coefficient

*Spearman’s rank correlation coefficient

**P < 0.001

### Validity

The result of the KMO test was 0.788 and Bartlett’s test of sphericity was 716.2 (df = 15, *P* < 0.001). These results suggested that EFA of the sample was appropriate. Table 3 shows the results of the EFA for the SPQ. The factor loadings of all items had exceeded the recommended 0.40. A one-factor model, accounted for 74.8% of the explanatory variance, was retained.

**Table 3:** Range, mean scores, corrected item-total correlations and factor analysis results for the SPQ

Item	Mean (SD)	Corrected item-total correlation	Cronbach’s alpha if item deleted	Factor loading
1. the support that I get from the people around me	2.25 (1.16)	0.870	0.902	0.922
2. the advice that I get from the people around me	2.15 (0.90)	0.839	0.906	0.880
3. how much opportunity I have to discuss the pain with the people around me	2.31 (1.17)	0.729	0.925	0.880
4. how much care I receive	1.99 (0.83)	0.749	0.918	0.875
5. how much understanding the people around me show	2.21 (0.84)	0.809	0.911	0.820
6. the practical help people around me give	2.09 (0.85)	0.803	0.912	0.805

In term of convergent validity, it was assessed by correlating the SPQ scores with the score of the global oral health question. The result showed that the correlation was good (Table 2).

## Discussion

In the present study, the translation and validation of the Chinese version of SPQ is carried out. Statistical analysis showed that the 6-item Chinese version of SPQ with one-factor structure was reliable and valid. It can be used in Chinese population to evaluate the patient’s satisfaction with pain-related social support.

In the current study, chronic TMD pain lasting for at least 3 months was used as a temporal criterion to recruit patients suffering from painful TMD. This was consistent with other previous studies [15, 26]. In order to get a semantically and conceptually similar version to the original SPQ, the process of translation and cross-cultural adaptation of the original SPQ was carried out. The Chinese version of SPQ was overall well received by the patients, and no problem or language difficulty existed.

In terms of reliability, both the internal consistency and test-retest reliability were proved to be good. The Cronbach’s alpha for the SPQ was 0.926, indicating a high level of internal consistency. This value indicates high correlations among items of SPQ. And when each item was deleted, the values of Cronbach’s alpha remained stable. The value of ICC (0.784, 90% CI: 0.648–0.893) was higher than the values in the original study, indicating good test-retest reliability [15]. For the selection of the time interval, previous study suggested that a period of 2 days to 2 weeks was thought to be suitable [27]. A period of 2 weeks was chosen in the present study. And in the original study, a maximal time interval of 8 weeks was adopted. The lower ICC value may be explained by the longer time interval in the original study [15].

Regarding factorial structure of the SPQ, the finding of EFA identified a one-factor structure of the SPQ. This result was consistent with previous study proposed by Van Der Lugt et al. [15]. Similar as the original study, the factor loadings of all items were above 0.80 [15]. As regards convergent validity, the Chinese version of SPQ had good correlation with the global oral health question. The finding was close to the values in the original study [15]. Overall, the Chinese version of SPQ had adequate validity for using in patients with painful TMD.

However, the present study has two limitations which requiring further assessment. Firstly, all patients were enrolled from a single university-affiliated hospital, and thus may not be able to represent all Chinese population affected by painful TMD. Secondly, the current study does not contain long-term follow-up analyses, and therefore the sensitivity and responsiveness of the Chinese version of SPQ could not be evaluated.

## Conclusion

The present study supports that the Chinese version of Social support and Pain Questionnaire (SPQ) can be used as a reliable and valid tool for Chinese patients with painful TMD.

## Abbreviations

EFA: Exploratory factor analysis
ICC: Intraclass correlation coefficient
KMO: Kaiser–Meyer–Olkin test
RDC/TMD: Research diagnostic criteria for temporomandibular disorders
SPQ: The Social support and Pain Questionnaire
TMD: Temporomandibular disorders

## References

[1] Ozdemir-Karatas M, Peker K, Balik A, Uysal O, Tuncer EB (2013). Identifying potential predictors of pain-related disability in Turkish patients with chronic temporomandibular disorder pain. J Headache Pain.

[2] Wu N, Hirsch C (2010). Temporomandibular disorders in German and Chinese adolescents. J Orofac Orthop.

[3] Deng YM, Fu MK, Hagg U (1995). Prevalence of temporomandibular joint dysfunction (TMJD) in Chinese children and adolescents. A cross-sectional epidemiological study. Eur J Orthod.

[4] Gil-Martinez A, Grande-Alonso M, Lopez-de-Uralde-Villanueva I, Lopez-Lopez A, Fernandez-Carnero J, La Touche R (2016). Chronic Temporomandibular disorders: disability, pain intensity and fear of movement. J Headache Pain.

[5] Suvinen TI, Reade PC, Kemppainen P, Kononen M, Dworkin SF (2005). Review of aetiological concepts of temporomandibular pain disorders: towards a biopsychosocial model for integration of physical disorder factors with psychological and psychosocial illness impact factors. Eur J Pain.

[6] Almoznino G, Zini A, Zakuto A, Sharav Y, Haviv Y, Hadad A, Chweidan H, Yarom N, Benoliel R (2015). Oral health-related quality of life in patients with Temporomandibular disorders. J Oral Facial Pain Headache.

[7] Tjakkes GH, Reinders JJ, Tenvergert EM, Stegenga B (2010). TMD pain: the effect on health related quality of life and the influence of pain duration. Health Qual Life Outcomes.

[8] He S, Wang J, Ji P (2016). Validation of the Tampa scale for Kinesiophobia for Temporomandibular disorders (TSK-TMD) in patients with painful TMD. J Headache Pain.

[9] Evers AW, Kraaimaat FW, Geenen R, Jacobs JW, Bijlsma JW (2003). Pain coping and social support as predictors of long-term functional disability and pain in early rheumatoid arthritis. Behav Res Ther.

[10] Ledoux E, Dubois JD, Descarreaux M (2012). Physical and psychosocial predictors of functional trunk capacity in older adults with and without low back pain. J Manip Physiol Ther.

[11] Romano JM, Jensen MP, Schmaling KB, Hops H, Buchwald DS (2009). Illness behaviors in patients with unexplained chronic fatigue are associated with significant other responses. J Behav Med.

[12] Kornblith AB, Herndon JE, Zuckerman E, Viscoli CM, Horwitz RI, Cooper MR, Harris L, Tkaczuk KH, Perry MC, Budman D, Norton LC, Holland J, Cancer, & Leukemia Group, B (2001). Social support as a buffer to the psychological impact of stressful life events in women with breast cancer. Cancer.

[13] Kristensen MT (2013). Hip fracture-related pain strongly influences functional performance of patients with an intertrochanteric fracture upon discharge from the hospital. PM R.

[14] Romano JM, Turner JA, Jensen MP, Friedman LS, Bulcroft RA, Hops H, Wright SF (1995). Chronic pain patient-spouse behavioral interactions predict patient disability. Pain.

[15] Van Der Lugt CM, Rollman A, Naeije M, Lobbezoo F, Visscher CM (2012). Social support in chronic pain: development and preliminary psychometric assessment of a new instrument. J Oral Rehabil.

[16] van den Akker-Scheek I, Stevens M, Spriensma A, van Horn JR (2004). Groningen Orthopaedic social support scale: validity and reliability. J Adv Nurs.

[17] Suurmeijer TP, Doeglas DM, Briancon S, Krijnen WP, Krol B, Sanderman R, Moum T, Bjelle A, Van Den Heuvel WJ (1995). The measurement of social support in the 'European research on incapacitating diseases and social Support': the development of the social support questionnaire for transactions (SSQT). Soc Sci Med.

[18] Kerns RD, Turk DC, Rudy TE (1985). The West Haven-Yale Multidimensional pain inventory (WHYMPI). Pain.

[19] Eklund A, Bergstrom G, Bodin L, Axen I (2016). Do psychological and behavioral factors classified by the West Haven-Yale Multidimensional pain inventory (Swedish version) predict the early clinical course of low back pain in patients receiving chiropractic care?. BMC Musculoskelet Disord.

[20] Turner RJ, Marino F (1994). Social support and social structure: a descriptive epidemiology. J Health Soc Behav.

[21] Schiffman E, Ohrbach R, Truelove E, Look J, Anderson G, Goulet JP, List T, Svensson P, Gonzalez Y, Lobbezoo F, Michelotti A, Brooks SL, Ceusters W, Drangsholt M, Ettlin D, Gaul C, Goldberg LJ, Haythornthwaite JA, Hollender L, Jensen R, John MT, De Laat A, de Leeuw R, Maixner W, van der Meulen M, Murray GM, Nixdorf DR, Palla S, Petersson A, Pionchon P, Smith B, Visscher CM, Zakrzewska J, Dworkin SF, International Rdc/Tmd Consortium Network, I. a. f. D. R., & Orofacial Pain Special Interest Group, I. A. f. t. S. o. P (2014). Diagnostic criteria for Temporomandibular disorders (DC/TMD) for clinical and research applications: recommendations of the international RDC/TMD consortium network* and Orofacial pain special interest Groupdagger. J Oral Facial Pain Headache.

[22] Terwee CB, Bot SD, de Boer MR, van der Windt DA, Knol DL, Dekker J, Bouter LM, de Vet HC (2007). Quality criteria were proposed for measurement properties of health status questionnaires. J Clin Epidemiol.

[23] Guillemin F, Bombardier C, Beaton D (1993). Cross-cultural adaptation of health-related quality of life measures: literature review and proposed guidelines. J Clin Epidemiol.

[24] Bartlett MS (1950). Tests of significance in factor analysis. Br J Stat Psychol.

[25] Peter MF, David M (2000). Quality of life assessment, analysis and interpretation.

[26] Matos M, Bernardes SF (2013). The Portuguese formal social support for autonomy and dependence in PAIN inventory (FSSADI_PAIN): a preliminary validation study. Br J Health Psychol.

[27] Marx RG, Menezes A, Horovitz L, Jones EC, Warren RF (2003). A comparison of two time intervals for test-retest reliability of health status instruments. J Clin Epidemiol.

